# Editorial: High color purity boron-based OLED materials

**DOI:** 10.3389/fchem.2024.1441517

**Published:** 2024-06-13

**Authors:** Jang Hyuk Kwon

**Affiliations:** Organic Optoelectronic Device Lab (OODL), Department of Information Display, Kyung Hee University, Seoul, Republic of Korea

**Keywords:** organic light emitting devices/display (OLED), boron, color purity, narrow spectra linewidth, efficiency

Since Tang and VanSlyke (1987)’s breakthrough with the first organic light-emitting diode (OLED), these devices have been thoroughly researched for real-world displays. Their appeal lies in their lightweight build, wide viewing angles, rapid response times, easy chemical customization of emitting molecules, low energy usage, compatibility with flexible plastic substrates, and adaptability to diverse display forms. Moreover, pure organic materials are vital in OLEDs, significantly influencing their luminescence. Recent advancements in OLEDs have shown significant improvements in efficiency and lifespan (Chan et al., 2021; Vasilopoulou et al., 2021; Naveen et al., 2022). Still, the broad emission spectra of materials pose challenges for achieving the high color purity required for future high-end displays like UHDTV. The BT 2020 color gamut standard mandates narrowband emissions for red, green, and blue, which is essential for enhancing color purity (Vasilopoulou et al., 2021). Recently, boron-based multi-resonant (MR) narrowband emitters have exhibited high color purity and significant potential in OLED applications, offering promising alternatives for improved OLED performance (Madayanad Suresh et al., 2020; Kim and Yasuda, 2022; Mamada et al., 2024).

This Research Topic comprises four submissions, each focusing on distinct aspects. Three submissions explore boron-based materials for OLED applications, likely investigating synthesis, characterization, and performance evaluation to enhance color purity and overall efficiency. Conversely, one article in the Research Topic focuses on designing and synthesizing a new [1 + 4+2] framework of multilayer targets with detailed photophysical characterizations. Xiaofeng et al. delved into the “Sterically-wrapped MR fluorophores” concept for quench-resistant and narrowband OLED applications. Their exploration included a thorough analysis of reported materials and their device performances. Additionally, they guided various approaches for achieving stable ultra-wide color gamut OLED displays. Gawale et al. elaborated into hyperfluorescence-based stable OLEDs, focusing primarily on material design, a deeper understanding of energy transfer mechanisms, and examining previously reported hyperfluorescence devices. Their exploration provides insights into the prospects for the display industry, highlighting the importance of innovative material design and a comprehensive understanding of energy transfer processes for advancing OLED technology. Keshri et al. introduced a novel Se-doped emitter, SeBSe, characterized by a helically distorted structure and a high reverse intersystem crossing (RISC) rate surpassing 108 s-1, resulting in a narrowband sky blue emission. Remarkably, OLEDs fabricated using the SeBSe material achieved a maximum external quantum efficiency of 9.3%. Zhang et al. successfully synthesized a new [1 + 4+2] multilayer framework employing modified dual Suzuki–Miyaura cross-couplings and group-assisted purification (GAP) chemistry. Comprehensive characterization via spectroscopic analysis and X-ray crystallography was conducted, along with detailed investigations into physical properties, including aggregation-induced emission (AIE).

This Research Topic is truly a testament to the collective dedication of researchers in unraveling the complexities of luminescent materials, particularly boron-based materials. The dynamic nature of this field underscores the relentless pursuit of knowledge and the collaborative spirit that propels scientific advancement forward in OLEDs. By laying the groundwork for future investigations, this work not only expands our understanding of chemistry but also holds promise for addressing pressing societal challenges in the display industries. It exemplifies the synergy that arises from the exchange of ideas and the shared commitment to pushing the boundaries of science for the betterment of humanity.

The gratitude extended to the contributors of this Research Topic is heartfelt and well-deserved. Their unwavering dedication and groundbreaking contributions have significantly enriched our collective understanding of high color purity materials, opening doors to future innovations, particularly in OLEDs. Their diverse insights and findings illuminate the multifaceted nature of chemistry’s pursuit to harness the potential of these compounds, offering exciting new avenues for discovery and application. It is through their collaborative efforts that we continue to push the boundaries of scientific exploration and pave the way for transformative advancements in boron-based narrowband materials.
